# Evaluation of the Psychometric Properties and Applicability of Agni Assessment Tools in Ayurveda: Protocol for a Systematic Review

**DOI:** 10.2196/79580

**Published:** 2026-06-18

**Authors:** Anjana Roy, Aadithyaraj KT, Shivakumar S Harti, Shibna MV, Yogesh Badwe

**Affiliations:** 1 Department of Swasthavritta & Yoga, All India Institute of Ayurveda New Delhi India; 2 Department of Shalya Tantra, All India Institute of Ayurveda New Delhi India

**Keywords:** Agni, Agnibala, Ayurvedic digestive fire, validity, reliability, psychometric properties, COSMIN, systematic review protocol

## Abstract

**Background:**

*Agni* (digestive or metabolic factors) is a fundamental concept in Ayurveda that governs digestion, metabolism, and absorption. The strength of *Agni* varies from individual to individual, depending on various factors. Given its clinical importance, several researchers have developed screening tools to estimate *Agni* strength. Identifying valid and reliable *Agni* assessment tools will enhance diagnostic precision. In research, standardized and validated instruments facilitate reproducible studies, enabling meaningful comparisons across populations and settings.

**Objective:**

This study aims to critically appraise and synthesize the best available evidence on screening instruments for assessing the strength of *Agni*, including both self-reported questionnaires and clinician-administered tools. This review will evaluate their psychometric properties, specifically focusing on content validity, construct validity, criterion validity, internal consistency, and test–retest reliability. Additionally, the review will assess the feasibility, efficiency, and applicability of these instruments in clinical and research settings within the framework of Ayurveda.

**Methods:**

In this proposed study, a systematic review will be conducted using the intended search strategy, using databases such as PubMed, EBSCO, Cochrane, Google Scholar (first 200 studies), the AYUSH research portal, and Digital Helpline for Ayurveda Research Articles. This study will focus on adults aged 18 years and older. Two independent reviewers will screen titles, abstracts, and full-text articles for eligibility and extract data on tool characteristics, psychometric properties, and applicability, with discrepancies resolved by a third reviewer. The psychometric quality of the included studies will be evaluated using QUADAS-2 (Quality Assessment of Diagnostic Accuracy Studies-2) and COSMIN (Consensus-based Standards for the Selection of Health Measurement Instruments). Extracted data will be synthesized using a narrative approach, supported by tabular summaries.

**Results:**

This systematic review was formally initiated in October 2025, following a planning phase that commenced in January 2025. Data from studies that meet the inclusion criteria will be systematically extracted, organized, and analyzed. The risk of bias and the overall quality of evidence will be evaluated, and the final review is expected to be completed and published by November 2026.

**Conclusions:**

This systematic review protocol outlines an approach to identify valid and reliable *Agni* assessment tools that contribute to the standardization of Ayurvedic diagnostics, enabling meaningful integration with contemporary biomedical research and highlighting the importance of transparency and adaptability in the field of Ayurveda. By establishing a structured evidence base, it aims to support the development of more robust, clinically relevant, and universally applicable assessment practices.

**Trial Registration:**

PROSPERO CRD42024626327; https://www.crd.york.ac.uk/PROSPERO/view/CRD42024626327

**International Registered Report Identifier (IRRID):**

DERR1-10.2196/79580

## Introduction

*Agni* in Ayurveda represents the vital force responsible for the digestion, metabolism, and transformation of substances in the human body, deeply embedded within ancient Indian medical philosophy and practice [1]. This concept is regarded as the cornerstone of health and disease, shaping a holistic understanding of the physiological balance and pathological disruptions through its nuanced roles: *Jataragni* (the digestive “fire” or “heat” principle), *Dhatwagni* (tissue-level metabolism), and *Bhutagni* (elemental metabolism) [2].

Some contemporary authors have proposed tentative correspondences between the concept of *Agni* and metabolic stages recognized in biochemistry. While *Jataragni* is analogous to enzymatic digestion (hydrolysis), which governs digestive enzymes, the mechanical breakdown of food, and absorption in the gastrointestinal tract, *Dhatwagni* corresponds to intermediary metabolism, which relates to tissue-specific metabolic activities (eg, liver, muscle) that transform absorbed nutrients for tissue building and energy. *Bhutagni* represents cellular metabolism (Krebs cycle). It reflects molecular processing at the cellular level, including mitochondria-driven metabolic pathways [3]. However, these remain hypothetical and require empirical testing.

Given the centrality of *Agni* in maintaining health, it becomes crucial to evaluate and assess its function accurately in clinical practice. Various *Agni* assessment tools have been developed by several researchers to gauge its state and influence on health. The initial search conducted for the published literature yielded several articles. The existing *Agni* assessment tools encompass questionnaires, clinical evaluations, and specific interventions, yet each face notable methodological and practical limitations that warrant systematic review. The diversity and developmental stages of these tools reflect ongoing efforts to operationalize the nuanced concept of *Agni* for clinical and research contexts. Yet, issues of standardization and psychometric rigor remain unresolved.

Among these, the Scale on Assessment of *Agni,* developed by Patil et al [4], is a physician-administered scale that assess digestive strength by administering a specific amount of ghee. Another tool for the assessment of *Agni* was developed by Eswaran et al [5]. It is an extensive questionnaire based on classical Ayurveda texts. The questionnaire employs a 5-point Likert scale for each question. One more *Agni* assessment tool, developed by Singh et al [6], has been used extensively in the field. It is a self-administered questionnaire aligning features with the 4 states of *Jataragni.* These existing instruments vary widely in the administration mode, length, and reported psychometric quality, with common gaps in content validity, factor structure, and feasibility testing.

Given these limitations and the central role of *Agni* in Ayurveda, a systematic review evaluating the psychometric properties, content clarity, and applicability of existing *Agni* assessment tools is essential to advance evidence-based clinical and research practice. This protocol outlines a rigorous approach to identify, appraise, and synthesize the validity, reliability, and practical utility of available tools, thereby addressing a critical gap and guiding the development of standardized, contextually relevant instruments that can strengthen interdisciplinary collaboration and clinical application. No previous systematic review of psychometric evidence for *Agni* assessment tools has been registered in PROSPERO.

## Methods

### Study Design

This protocol is prepared and reported using the PRISMA-P (Preferred Reporting Items for Systematic Review and Meta-Analysis—Protocol) [7] guidelines and Cochrane Handbook for Systematic Reviews of Interventions [8]. In addition, registration in PROSPERO has been completed for the upcoming trial (CRD42024626327). Searching, selection of the studies, quality assessment, and data abstraction of the selected studies will be done independently by two reviewers. PubMed, EBSCO, Cochrane, Google Scholar (first 200 studies), AYUSH research portal, and Digital Helpline for Ayurveda Research Articles databases will be searched using relevant search strategies. The STARD (Standards for the Reporting of Diagnostic Accuracy Studies) guidelines will be used while conducting the systematic review of the selected studies [9]. Retrieved studies will be evaluated for relevance (by considering if the psychometric data were reported) and quality by using tools such as COSMIN (Consensus-based Standards for the Selection of Health Measurement Instruments) [10] and QUADAS-2 (Quality Assessment of Diagnostic Accuracy Studies-2) [11].

### Eligibility Criteria for Study Selection

Studies will be included if they report at least one psychometric property (eg, content, construct, or criterion validity; internal consistency, test-retest, or inter-rater reliability) of *Agni* assessment tools and involve participants aged 18 years or older, encompassing both healthy and diseased populations, in any setting without specific restrictions. Only studies published in English with full-text availability will be considered. Qualitative studies, review articles, and gray literature will be excluded to ensure focus on empirical psychometric data, and non-English publications will be excluded, as they do not represent the primary language of communication in the relevant scientific literature.

### Search Strategy

The search strategy was developed through an iterative and systematic process to ensure the comprehensive identification of relevant studies. An initial limited search was conducted to identify key terms and phrases appearing in the titles, abstracts, and subject descriptors of pertinent literature. These terms were analyzed to refine and expand the list of keywords and controlled vocabulary. Subsequently, Medical Subject Headings (MeSH) and equivalent indexing terms were incorporated to improve the search's accuracy and sensitivity.

The preliminary list of search terms such as *Agni*, *Agnibala*, digestive function, Ayurveda, psychometric, reliability, and validity, was pilot tested in PubMed to assess relevance and comprehensiveness. Based on the initial results, the search syntax was refined by applying Boolean operators (AND, OR) and adjusting truncation and proximity operators as appropriate. The final strategy was reviewed and validated in consultation with an academic librarian experienced in systematic reviews to ensure optimal database coverage and search precision.

### Selection of Studies

**Figure 1:**
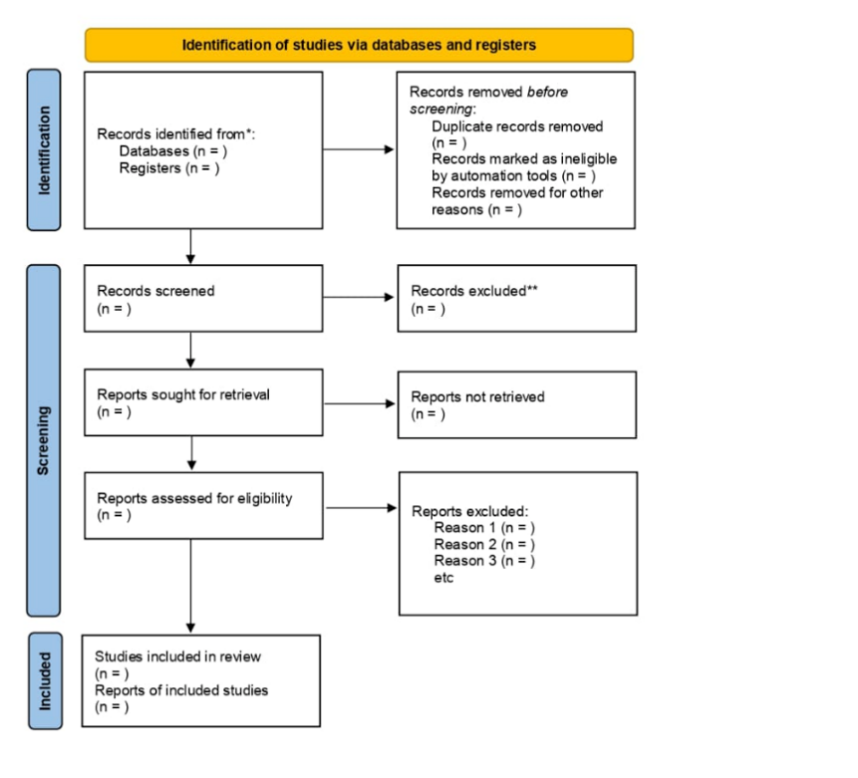
PRISMA (Preferred Reporting Items for Systematic Review and Meta-Analysis) 2020 flow diagram * Consider, if feasible to do so, reporting the number of records identified from each database or register searched (rather than the total number across all databases/registers) ** If automation tools were used, indicate how many records were excluded by a human and how many were excluded by automation tools [12].

Two reviewers (AR and AKT) will independently screen the titles and abstracts based on the established inclusion and exclusion strategies. Where the abstract does not provide enough information or the reviewers are not in agreement, the full-text articles will be reviewed. Any discrepancies that arise will be resolved by consensus or consultation with a third reviewer (SMV or SSH or YB). Studies without psychometric evaluation or articles without full-text availability will be excluded from the review. Studies will be classified as include, exclude, and unclear after reviewing article titles and abstracts to determine their eligibility. After screening the title and abstract, the included studies will be retrieved with its full text to evaluate the study in detail. The selection of studies will follow the PRISMA guidelines [12]. The PRISMA 2020 flow diagram (Figure 1) will be used to present the particulars of the selection process.

### Data Collection Process

For the systematic review, the extraction of data will be performed independently by 2 reviewers (AR and AK). A third reviewer (SMV or SSH or YRB) will address and reconcile all the inconsistencies. Data will be extracted from the articles included in the review using the standardized data extraction form that will be designed by the researcher. The extracted data will include information such as the name of the authors, year of publication, population, gold standard, study settings, sample size, psychometric properties (validity and reliability) of the screening instruments, and any other relevant details, adhering to the standards described in the STARD guidelines. All results will be subjected to double data entry by the 2 reviewers.

### Outcome Measures

The outcome measures are to identify *Agni* assessment instruments according to the quality of evidence supporting their measurement properties using COSMIN and QUADAS-2 criteria and to provide evidence-based recommendations for clinical and research use.

### Assessment of Risk of Bias

The risk of bias in the included studies will be evaluated using tools such as COSMIN and QUADAS-2. The methodological quality of the included studies will be assessed using the COSMIN checklist, which evaluates the risk of bias related to the measurement properties of the selected *Agni* assessment tools. COSMIN defines 3 overarching quality domains: reliability, validity, and responsiveness [13]. The reliability domain reflects the extent to which the measurement is free from error and consists of 3 measurement properties—internal consistency, reliability, and measurement error. The validity domain examines the degree to which the instrument accurately measures the intended construct and encompasses 5 measurement properties: content validity, construct validity (including structural validity, hypothesis testing, and cross-cultural validity), and criterion validity. The responsiveness domain, comprising the single measurement property known as responsiveness, assesses the ability of an outcome measure to detect change over time in the construct being measured. In accordance with COSMIN guidelines, each measurement property will be rated using the 4-point scoring system (very good, adequate, doubtful, or inadequate), and the “worst score counts” principle will be applied, whereby the lowest rating among the items within a measurement property determines the overall risk-of-bias judgement for that property [14].

The QUADAS-2 assessment will examine four key domains: (1) Patient Selection to describe methods of patient selection, (2) Index Test to assess whether the *Agni* assessment tool was applied in a standardized manner, (3) Reference Standard to determine if the reference standard used for validation is reliable and independent of the index test, and (4) Flow and Timing to Check if all participants underwent the same index test and reference standard and whether any delays could introduce bias [15]. The risk of bias and applicability judgments will be categorized as high, low, or unclear based on assessments across the 4 domains. The findings from this will be illustrated as “RoB summary” and “RoB graph” [16].

### Data Synthesis and Analysis

A narrative synthesis will be conducted due to anticipated heterogeneity in study designs, populations, and psychometric evaluation methods, which limits the feasibility of meta-analysis. Data will be descriptively summarized and organized under four domains: (1) Study Characteristics detailing authors, year, setting, population, and sample size; (2) Tool Characteristics including type of tool, scoring system, and mode of administration; (3) Psychometric Properties such as validity (face, content, construct, criterion) and reliability (test–retest, inter-rater, internal consistency); and (4) Applicability and Usability outlining study context (clinical, research, or community) and feasibility in Ayurvedic practice. To address heterogeneity, subgroup analyses will be explored by tool type, study setting, and population characteristics. Findings will be presented in summary tables, figures, and descriptive text, providing a structured overview of tool performance and psychometric quality.

## Results

The systematic review was formally initiated in October 2025, following a planning phase that commenced in January 2025. Data from eligible studies will be systematically extracted, tabulated, and narratively synthesized to capture tool characteristics, psychometric performance, and practical applicability. Results will be presented in detailed tables and figures that illustrate risk of bias assessments to facilitate interpretation. The final review, including data analysis and synthesis, is expected to be completed and published by November 2026.

## Discussion

This systematic review protocol, prepared in accordance with the PRISMA-P guidelines and the Cochrane Handbook for Systematic Reviews of Interventions, delineates a methodical approach to evaluating the psychometric properties and clinical applicability of existing *Agni* assessment instruments. Within the Ayurvedic framework, *Agni* constitutes a fundamental determinant of physiological equilibrium, metabolic efficiency, and overall health maintenance. Its proper functioning is considered essential for maintaining homeostasis and preventing pathological manifestations, whereas its derangement is a primary etiological factor in disease development [17]. Traditional approaches to *Agni* assessment emphasize comprehensive clinical observation, incorporating parameters such as appetite, food digestibility, excretory patterns, bodily vitality, complexion, and mental clarity [18]. In recent decades, scientific advancements have enabled the integration of traditional Ayurvedic observations with objective biomedical assessments, including metabolic profiling, enzymatic assays, calorimetric evaluation, and metabolic imaging techniques [19]. This confluence of traditional and contemporary methodologies has led to the development of quantitative *Agni* assessment scales that endeavor to align subjective Ayurvedic evaluations with measurable physiological correlates. The validity and reliability of these assessment tools are crucial to ensuring diagnostic consistency, research integrity, and therapeutic precision within Ayurveda. Despite their proliferation, there has been no systematic synthesis of evidence evaluating the psychometric soundness of these instruments, specifically their validity, reliability, responsiveness, and cross-cultural adaptability. Thus, this protocol aims to systematically identify, appraise, and synthesize available evidence on *Agni* assessment tools to determine their theoretical foundation, measurement quality, and empirical credibility. By adhering to a transparent, rigorous methodological framework, this review might provide a comprehensive evidence base to support the refinement, validation, and integration of *Agni* assessment scales in contemporary Ayurvedic research and clinical practice.

Further, the use of standardized evaluation frameworks such as COSMIN and QUADAS-2 will facilitate a structured appraisal of methodological quality, minimize bias, and enhance the reliability of findings. The COSMIN and QUADAS-2 frameworks were selected for their robust criteria in evaluating psychometric properties and diagnostic accuracy, respectively. COSMIN will assess the methodological quality of studies reporting validity and reliability, while QUADAS-2 will evaluate diagnostic tool performance, particularly addressing challenges such as the absence of a universal gold standard for *Agni* and variability in tool administration across diverse settings.

A key anticipated challenge in conducting this systematic review will be the potential heterogeneity among available studies in terms of design, population characteristics, and assessment procedures used across various *Agni* evaluation tools. Given that *Agni* assessment is inherently rooted in Ayurvedic theoretical constructs, differences in the interpretation, operationalization, and measurement of *Agni* parameters may impede direct comparisons and meta-analytic synthesis. Furthermore, the absence of a universally recognized gold standard for *Agni* assessment poses limitations in establishing criterion validity and benchmarking the psychometric performance of individual tools.

Beyond these primary concerns, several additional methodological and interpretive challenges may arise. Publication bias remains a notable risk, as studies with favorable psychometric results are more likely to be published, potentially inflating the perceived robustness of specific instruments. Similarly, limited generalizability may limit the applicability of the findings. Variability in the conceptual definitions of *Agni* across classical Ayurvedic texts, as well as in its practical interpretation by different schools of thought, can further contribute to inconsistencies in the study outcomes. Regional and linguistic differences in Ayurveda education and practice may also influence item formulation, response interpretation, and tool validation. Moreover, small sample sizes, limited cross-validation, and inadequate reporting of psychometric testing protocols may constrain the reliability of conclusions drawn from the existing evidence base.

To address these challenges, the review will adopt several mitigating strategies. Comprehensive and systematic search methods, along with rigorous inclusion and exclusion criteria, will be applied to ensure methodological consistency and comparability across studies. Where possible, subgroup analyses will be conducted to explore heterogeneity based on population demographics, geographic regions, and variations in Ayurvedic interpretation. In addition, explicit documentation of theoretical definitions and measurement models of *Agni* in each included study will be maintained to enhance transparency and facilitate conceptual synthesis. Upon completion of the systematic review, patient and public involvement representatives will review the plain-language summary to ensure cultural appropriateness and linguistic equity.

This systematic review will provide a comprehensive understanding of the methodological quality and empirical robustness of existing *Agni* assessment tools, addressing a critical gap in standardized diagnostic methods in Ayurveda. Standardizing *Agni* assessment has significant relevance for national digital health efforts, as validated tools can be meaningfully integrated into the Ayushman Bharat Digital Health Mission to support interoperable, patient-centered Ayurvedic health records. Building on the findings of this review, we also propose initiating a Delphi consensus process to establish minimum psychometric standards for future *Agni* assessment instruments, thereby promoting methodological rigor and consensus across the field. To ensure transparency and accessibility, the review results, datasets, and related materials will be shared through open-access publication and deposited on Zenodo. Finally, the outcomes of this review will inform the design of a subsequent empirical validation study using the top-ranked tool, advancing the development of reliable, clinically relevant, and widely applicable frameworks for *Agni* assessment in Ayurveda.

One of the limitations of this study is its restriction to English-language publications. While this may lead to the exclusion of relevant evidence, the studies published in English will be chosen to ensure methodological consistency in data extraction, minimize the risk of misinterpretation of psychometric concepts during translation, and maintain feasibility, given the resource constraints for multilingual screening and appraisal.

## Abbreviations

COSMIN: Consensus-based Standards for the Selection of Health Measurement Instruments
MeSH: Medical Subject Headings
PRISMA-P: Preferred Reporting Items for Systematic Review and Meta-Analysis—Protocols
PRISMA: Preferred Reporting Items for Systematic Review and Meta-Analysis
QUADAS-2: Quality Assessment of Diagnostic Accuracy Studies-2
STARD: Standards for the Reporting of Diagnostic Accuracy Studies

## Data Availability

The datasets generated or analyzed during this study are available from the corresponding author on request.
